# Broadband Profiled Eye-Safe Emission of LMA Silica Fiber Doped with Tm^3+^/Ho^3+^ Ions

**DOI:** 10.3390/ma16247679

**Published:** 2023-12-17

**Authors:** Piotr Miluski, Krzysztof Markowski, Marcin Kochanowicz, Marek Łodziński, Wojciech A. Pisarski, Joanna Pisarska, Marta Kuwik, Magdalena Leśniak, Dominik Dorosz, Jacek Żmojda, Tomasz Ragiń, Jan Dorosz

**Affiliations:** 1 Faculty of Electrical Engineering, Bialystok University of Technology, Wiejska 45D Street, 15-351 Bialystok, Poland; krzysztof.markowski@sd.pb.edu.pl (K.M.); m.kochanowicz@pb.edu.pl (M.K.); j.zmojda@pb.edu.pl (J.Ż.); tomasz.ragin@pb.edu.pl (T.R.); doroszjan@pb.edu.pl (J.D.); 2 Faculty of Geology, Geophysics and Environment Protection, AGH University of Science and Technology, 30 Mickiewicza Av., 30-059 Krakow, Poland; lodzinsk@agh.edu.pl; 3 Institute of Chemistry, University of Silesia, 9 Szkolna Street, 40-007 Katowice, Poland; wojciech.pisarski@us.edu.pl (W.A.P.); joanna.pisarska@us.edu.pl (J.P.); marta.kuwik@us.edu.pl (M.K.); 4 Faculty of Materials Science and Ceramics, AGH University of Science and Technology, 30 Mickiewicza Av., 30-059 Krakow, Poland; mlesniak@agh.edu.pl (M.L.); ddorosz@agh.edu.pl (D.D.)

**Keywords:** MCVD, Large Mode Area Fiber LMA, broadband emission, Tm^3+^/Ho^3+^

## Abstract

LMA (Large Mode Area) optical fibers are presently under active investigation to explore their potential for generating laser action or broadband emission directly within the optical fiber structure. Additionally, a wide mode profile significantly reduces the power distribution density in the fiber cross-section, minimizing the power density, photodegradation, or thermal damage. Multi-stage deposition in the MCVD-CDT system was used to obtain the structural doping profile of the LMA fiber multi-ring core doped with Tm^3+^ and Tm^3+^/Ho^3+^ layer profiles. The low alumina content (Al_2_O_3_: 0.03wt%) results in low refractive index modification. The maximum concentrations of the lanthanide oxides were Tm_2_O_3_: 0.18wt % and Ho_2_O_3_: 0.15wt%. The double-clad construction of optical fiber with emission spectra in the eye-safe spectral range of (1.55–2.10 µm). The calculated LP_01_ Mode Field Diameter (MFD) was 69.7 µm (@ 2000 nm, and 1/e of maximum intensity), which confirms LMA fundamental mode guiding conditions. The FWHM and λmax vs. fiber length are presented and analyzed as a luminescence profile modification. The proposed structured optical fiber with a ring core can be used in new broadband optical radiation source designs.

## 1. Introduction

Currently, active optical fibers find a wider range of applications in open space applications. Fiber-optic radiation sources with high power density (including lasers and broadband radiation sources) operating in open space imply new safety requirements for their use. Exposure of the human body to a focused beam of radiation exposes us to the possibility of serious damage, hence the generally known protection requirements depending on the class of the laser device, depending on, among others, the power of laser radiation (international laser safety standard IEC 60825-1) [1,2,3]. Human eyesight is particularly vulnerable as the eye is a very delicate organ, and even a relatively low intensity of radiation can cause damage to it. This can range from ordinary eye strain to a glare effect much more significant than that caused by the sun to the final loss of visual acuity and up to complete loss of blindness [4,5,6,7,8,9]. Among the currently developed research directions, the eye-safe range with a spectral range above 1.4 µm seems especially attractive. This range is also characterized by relatively low attenuation by the atmosphere. The radiation in this spectral range is strongly absorbed in the cornea and lens of the eye and, therefore, cannot reach the much more sensitive retina. This calls for intensive research to develop fiber optic radiation sources at new wavelengths in the above range. Lasers and broadband sources are used, e.g., in remote sensing, LiDAR (Light Detection and Ranging), medicine, sensing systems, and free space telecommunication [10,11,12,13,14,15]. Due to the small diameters of optical fibers, there are significant power densities. Hence, the most common matrix for the fabrication of such sources is silica. It is characterized by a significant damage threshold of 0.2 J/cm^2^ at 1550 nm and low attenuation up to the spectral range of about 2.2 µm [16,17,18,19]. Production of silica optical fibers, due to high processing temperatures (softening point above 1700 °C), doping of silica is limited to inorganic compounds only. Germanium and aluminum are typically used to increase the refractive index, while lanthanide compounds are used for luminescence generation [20,21]. For the considered range of radiation (1.4–2.2 µm), Er^3+^, Tm^3+^, and Ho^3+^ ions can be used [20,22,23,24,25,26,27]. One of the most commonly used technologies for the production of silica optical fibers is the modified chemical vapor phase deposition (MCVD) method. This method has been developed since the 1970s. Initially, it was used for the production of telecommunications optical fibers. When active optical fibers (lanthanide-doped) were developed, they found applications in manufacturing optical amplifiers and lasers [28]. Currently, special attention is paid to new methods of doping. Hence, the developed doping technology using organometallic lanthanide compounds and the Chelate Doping Technology (CDT) assisted Plasma Chemical Vapor Deposition, introducing metallic nanoparticles, quantum dots, and nanocrystals [28,29,30,31,32]. Chemical Vapor Deposition technology supplies a chemical reaction zone to form silica inside an ultrapure quartz tube. The silica formation zone is defined by the distribution of the high-temperature area produced typically by a moving oxyhydrogen burner. In addition to the burner, a tube furnace may be used. In the presence of high temperatures, silica is formed while maintaining the proper flow of gases carrying reactants. The basic chemical reactions accompanying the formation of silica are presented in Formulas (1)–(4) [33].

SiCl_4_ + O_2_ → SiO_2_ + 2Cl_2_ (1)

GeCl_4_ + O_2_ → GeO_2_ + 2Cl_2_ (2)

4POCl_3_ + 3O_2_ → 2P_2_O_5_ + 6Cl_2_ (3)

4BCl_3_ + 3O_2_ → 2B_2_O_3_ + 6Cl_2_ (4)

**Figure 1:**
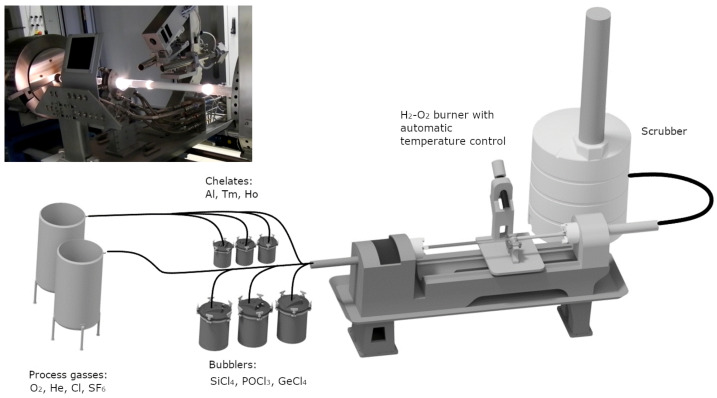
Schematic of Modified Chemical Deposition System (inset: photo of the system installed in the Bialystok University of Technology).

The proper composition of the glass-forming system is the basis for obtaining good optical properties of the preform. In addition to modifying the refractive index (RI), they affect the matrix phonon energy, phase separation, nonlinearity, photodarkening, and the level of rare earth doping. Therefore, a system of precise control of the chemical composition of the preform along its entire length is crucial. This was discussed in detail in prior publications [34,35,36,37,38,39,40]. The increasing refractive index of silica is performed using phosphorus, germanium, and aluminum. Significantly, the lanthanides increase RI. Historically, the first developed method of introducing active dopants (lanthanides) into the preform was the solution doping method, based on such temperature control to create a porous layer of silica inside the quartz tube. In the next stage, this structure was soaked in an ethanol solution of lanthanide, and after drying, the collapsing process. Due to the solution’s limited absorption and penetration depth, it enables the production of active optical fibers with relatively small core diameters. An alternative to this solution is the Chelate Doping Technology (CDT) method presented in Figure 1, which allows the delivery of lanthanides to the reaction zone in the form of organometallic compounds using carrier gases (typically helium). This allows the preform to be produced in one process, which minimizes the contamination with hydroxyl ions (OH^−^), the leading cause of absorption bands in the near-infrared range (typically in the range of 1.4 µm and above 1.7 µm). For the construction of optical fibers intended for operation in these spectral ranges, the OH^−^-content is required to be below a few ppm. On the other hand, silica fibers with a high content of hydroxyl groups (in the order of 600–1000 ppm) are used in the visible and ultraviolet range due to low attenuation in this range [41]. An undoubted advantage of the CDT technology is the possibility of profiling the refractive index and lanthanide doping profile, giving a wide range of developing new optical fiber constructions, including active optical fibers with a large core for laser applications and broadband radiation sources. A new trend in active optical fiber technology is the construction of novel LMA. They solve several problems occurring in classic constructions of step-index fibers, e.g., obtaining a high-quality M^2^ ≈ 1 output beam and maintaining single-mode operation with significant core diameters. The increase of the core structure also reduces the optical power density, thus minimizing non-linear effects disturbing the stable operation of laser systems, photodarkening effects, and the possibility of thermal damage [42,43,44].

One of the constructions of LMA fibers is the multi-ring structure of the refractive index profile that allows for increasing the effective propagation area of the fundamental mode (mode field diameter) even several hundred times [45,46,47,48]. In fact, there is a compromise between the ideal shape close to the Gaussian profile of the fundamental mode in classic construction single-mode fibers and increasing the mode field diameter (MFD) using multi-ring LMA construction fibers [49,50,51,52,53]. The multi-ring structures of the core profile were historically used for bend-resistant and dispersion-shifted/flattened optical fibers [54,55,56,57]. Complex constructions of fiber optic preforms consisting of different composition layers can be produced using the MCVD-CDT technology. Moreover, to obtain the assumed ratio of core-cladding diameters, preforms are often additionally stretched and overcladded. Among the fiber optic sources of radiation, constructions operating in the single-mode regime with emission in the range close to 1.8–2.1 µm are limited to doping with thulium and holmium ions. Above this range, the attenuation of the silica matrix increases significantly, which makes it difficult to build efficient systems of optical sources based on lanthanide emission. A significant advantage of this range is that it is easier to fulfill the conditions of single-mode operation for longer wavelengths, low attenuation of the atmosphere, and safety of use in the free space. In multilayer structures, it is possible to shape not only the modal properties but also the luminescence profile by selecting the arrangement of dopant layers and their concentrations, enabling a wide modification of these parameters. Typically, to obtain single-mode operation for optical fibers with large core diameters, the difference in the refractive indices of the core and the cladding ∆n is reduced and limits the level of doping with lanthanides, increasing the refractive index. Moreover, to increase the doping level, alumina is most often used additionally, which further increases its value. In this manuscript, we present a new construction of the LMA fiber with a core made of a multilayer structure doped with Tm^3+^ and Tm^3+^/Ho^3+^, which allows to obtain broadband emission in the range of 1.55–2.10 µm.

## 2. Materials and Methods

The fiber optic preform was fabricated using a modified vapor deposition technique called MCVD-CDT, which incorporated a doping system employing organometallic lanthanide compounds (Optacore). These lanthanide precursors (Tm(tmhd)_3_, Ho(tmhd)_3_, and AlCl_3_ were evaporated using helium as a carrier gas. Oxygen lines were used to transport silicon tetrachloride (SiCl_4_), phosphorus oxychloride POCl_3_, and sulfur hexafluoride SF_6_ to the Heraus (28/24 mm outer/inner diameter) substrate F300 tube. To control the deposition rates and hot zone parameters, a closed-loop temperature control system consisting of a burner-type pyrometer and mass flow controllers, was employed. The refractive index profile of the reform was measured using a P104 Photon Kinetics preform analyzer (He-Ne, at room temperature). The Control Interface drawing tower was used with a Centorr furnace equipped with a purified graphite heater and a heat shield assembly for the fiber drawing process. A precise control system of the drawing tower enables highly accurate management of important furnace parameters, including gas flow and temperature control, automatic fiber diameter control, and UV curing conditions. The outer cladding was created using UV-cured polymer cladding. The optical fiber was designed to be single-mode double-clad with a core/cladding diameter of 18/240 µm. Luminescence spectra of the preform (3 mm thickness discs) and fiber (cut-back method) were measured using a 796 nm laser diode operating in continuous wave mode (CW) with an output power of 3.1 W, and the Yokogawa AQ6375B Optical Spectrum Analyzer (OSA) in the 1.3–2.3 µm range. Structural characterization of the core structure (doping profiles) was performed using the Electron Probe Micro-Analyzer EMPA-WDS (@ 15 kV). The EPMA-WDS method was used to examine the doped system measured with an accuracy of 260 points in the cross-section of the preform core. Decay time measurements of Tm^3+^ and Ho^3+^ ions were carried out using the PTI QuantaMaster QM40 system. A tunable pulsed optical parametric oscillator (OPO) was pumped by the third harmonic of an Nd:YAG laser, specifically the Opolette 355 LD model. The laser system was equipped with a pair of monochromators (focal length 200 mm). The detection system was a Hamamatsu H10330B-75 detector, which was stored using a PTI ASOC-10 [USB-2500] oscilloscope. The optical attenuation was measured by the optical time-domain reflectometer at 1310 nm (2nd optical window). The simulation of modal properties and dispersion of the proposed optical fiber design was performed using the RP Fibre Power software.

## 3. Results

### 3.1. Optical Fiber Preform Characterization

**Figure 2:**
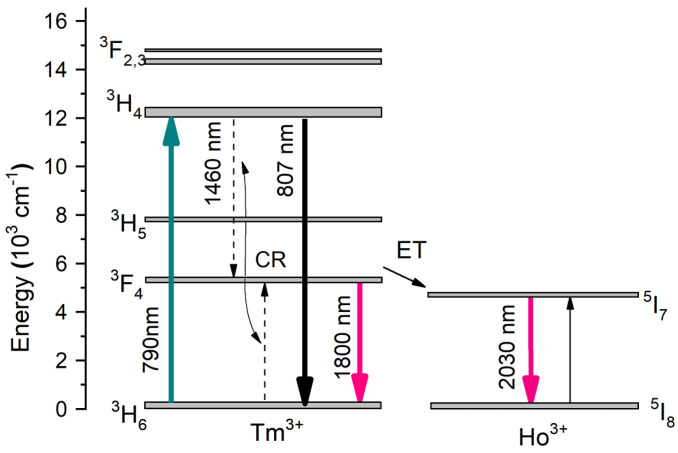
The energy level of Tm^3+^/Ho^3+^ co-doped silica (ET-energy transfer).

A diagram of energy levels with the main energy transitions of Tm^3+^ and Ho^3+^ marked is presented in Figure 2. Illustrations of energy level configurations depicting specific energy states of Tm^3+^ and Ho^3+^ ions, along with their corresponding 1.47 µm (^3^H_4_) and 2 µm (^5^I_7_) emission transitions, as well as the mechanisms for energy transfer can be observed [58]:

Tm^3+^ migration: Tm^3+^ (^3^F_4_, ^3^H_6_)→Tm^3+^ (^3^H_6_, ^3^F_4_) (5)

Tm^3+^→Ho^3+^ energy transfer: Tm^3+^ (^3^F_4_)→Ho^3+^ (^5^I_7_) (6)

Tm^3+^→Ho^3+^ backtransfer: Ho^3+^ (^5^I_7_)→Tm^3+^ (^3^F_4_) (7)

Tm^3+^ crossrelaxation: Tm^3+^ (^3^H_4_, ^3^H_6_)→Tm^3+^ (^3^F_4_, ^3^F_4_) (8)

^3^H_4_ Tm^3+^ migration: Tm^3+^ (^3^H_4_, ^3^H_6_)→Tm^3+^ (^3^H_6_, ^3^H_4_) (9)

The efficient excitation of thulium ions can be obtained by ground state absorption (GSA) excitation and the corresponding transition near 790 nm of ^3^H_6_→^3^H_4_ (Tm^3+^), which coincides well with the present well-developed and efficient construction of AlGaAs diode lasers is attractive for Tm^3+^ ions excitations. To enhance the efficiency of the Tm→Ho energy transfer process, a thorough investigation into the impact of each dopant ion’s concentration is crucial. For instance, the energy transfer to Ho^3+^ ions, which emit luminescence around 2 µm, making it suitable for various radiation sources emission in this spectral range. It is important to notice that the cross-relaxation mechanism is an efficient way to obtain two excited Tm^3+^ ions into the upper laser level ^3^F_4_ by only one pump photon and is an effective way to achieve population inversion in amplified spontaneous emission (ASE) and stimulated emission (LASER) structures.

**Figure 3:**
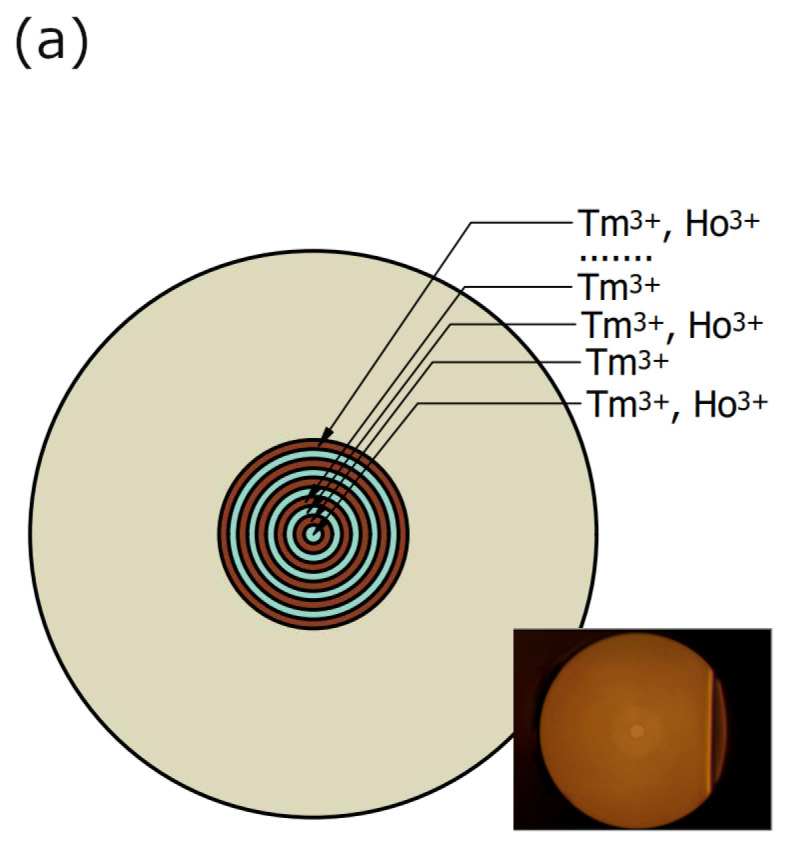

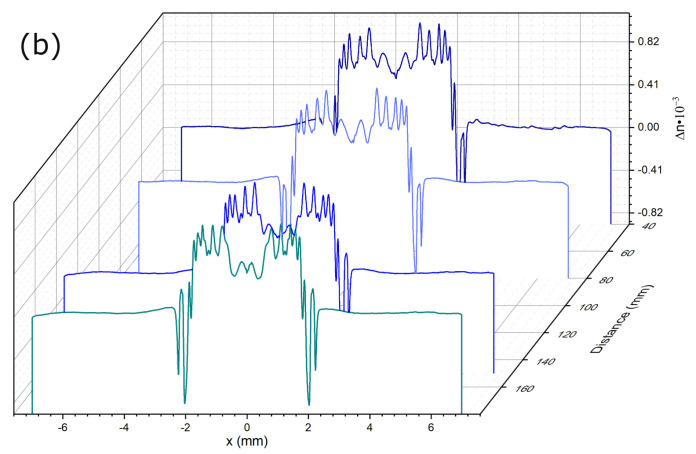
(**a**) The doping scheme of the preform, inset: photo of the fiber and (**b**) the refractive profile of the refractive index measured for 40, 80, 150, and 180 mm.

The fabrication of the preform, due to the assumed construction parameters (Tm^3+^ and Tm^3+^/Ho^3+^ layers) presented in Figure 3a and the relationship of core and cladding diameter, additionally required the process of stretching and jacketing on the MCVD lathes. To confirm the stability of the silica deposition parameters, the refractive profile was measured at a wavelength of 632.8 nm for selected positions in the fiber preform. The results are presented in Figure 3b. The recorded profiles are convergent and confirm the stability of the preform manufacturing process. The refractive index modification results from the doping profile (aluminum, thulium, and holmium). The low NA = 0.054 (Δn = 1 × 10^−3^) result in the calculated LP_01_ Mode Field Diameter (MFD) was 69.7 µm (@ 2000 nm).

**Figure 4:**
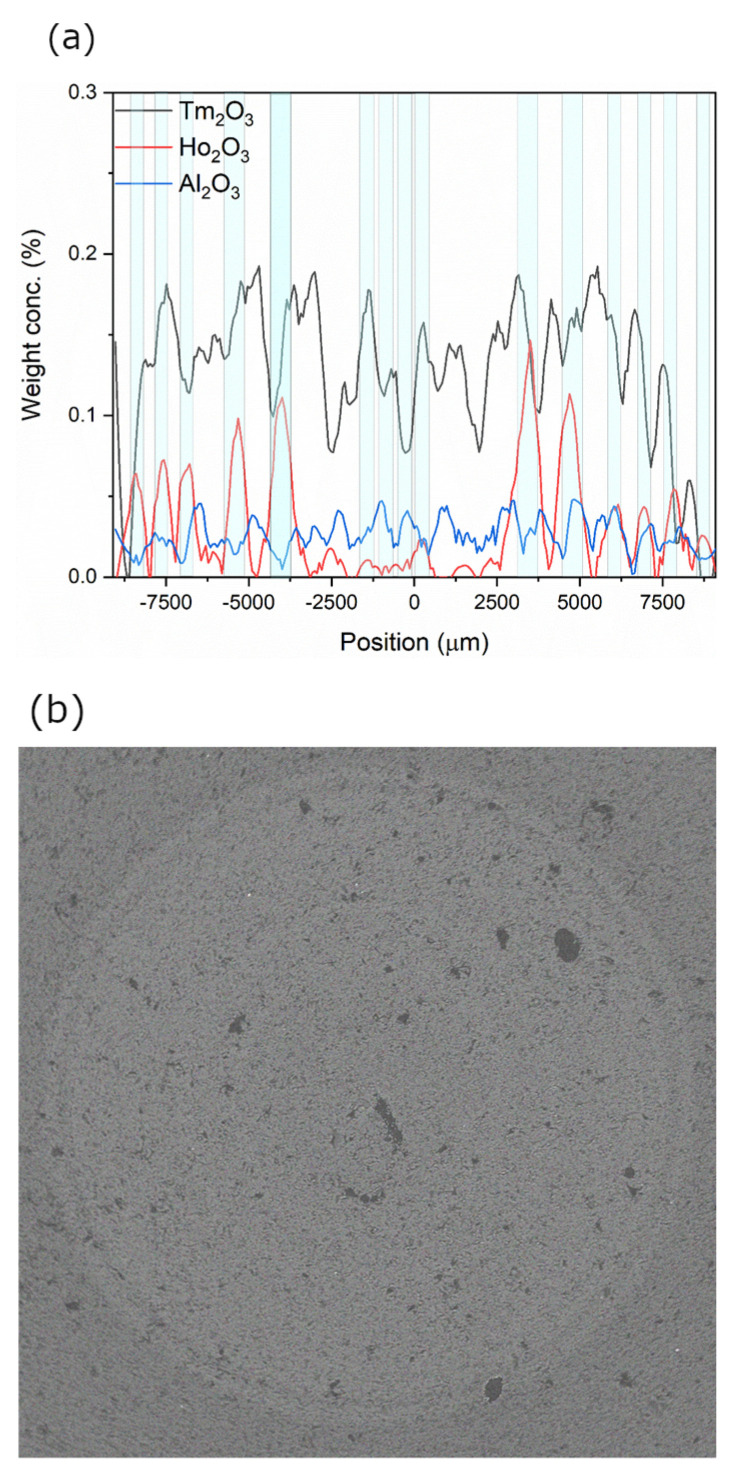
(**a**) The doping profile (EPMA-WDS) of the preform and (**b**) the SEM image of the fiber core region.

Maximum weight concentrations measured using EPMA-WDS (Figure 4a) were Al_2_O_3_: 0.03wt%, Tm_2_O_3_: 0.18wt%, and Ho_2_O_3_: 0.15wt% (Tm^3+^/Ho^3+^ co-doped layers marked as a background color change). The Scanning Electron Microscopy (SEM) image (Figure 4b) of the core region confirms the spatial distribution of dopants visible as brighter radial areas in the image. A cylindrical core structure with no apparent ellipticity is also visible. There are also no visible inhomogeneities in the individual layers forming the core, which confirms high deposition process stability. The SEM image also shows no phase separation phenomena in the optical fiber preform core and high-quality preform geometry.

The dispersion characteristics of fiber optics play an essential role in designing fiber optic communication systems. They determine the maximum possible data transfer in communication systems (so-called “eye diagram”). Development of telecommunications and optimization of dispersion characteristics based mainly on modifying the refractive profile (e.g., W or M type). From the point of view of non-communication applications of fiber optics, dispersion is often used in the development of radiation sources using the non-linear dependence of the refractive index of the fiber core area to generate a series of harmonics of excitation radiation (with significant power density) and obtain ultra-broadband emission of radiation generated directly in the fiber structure.

**Figure 5:**
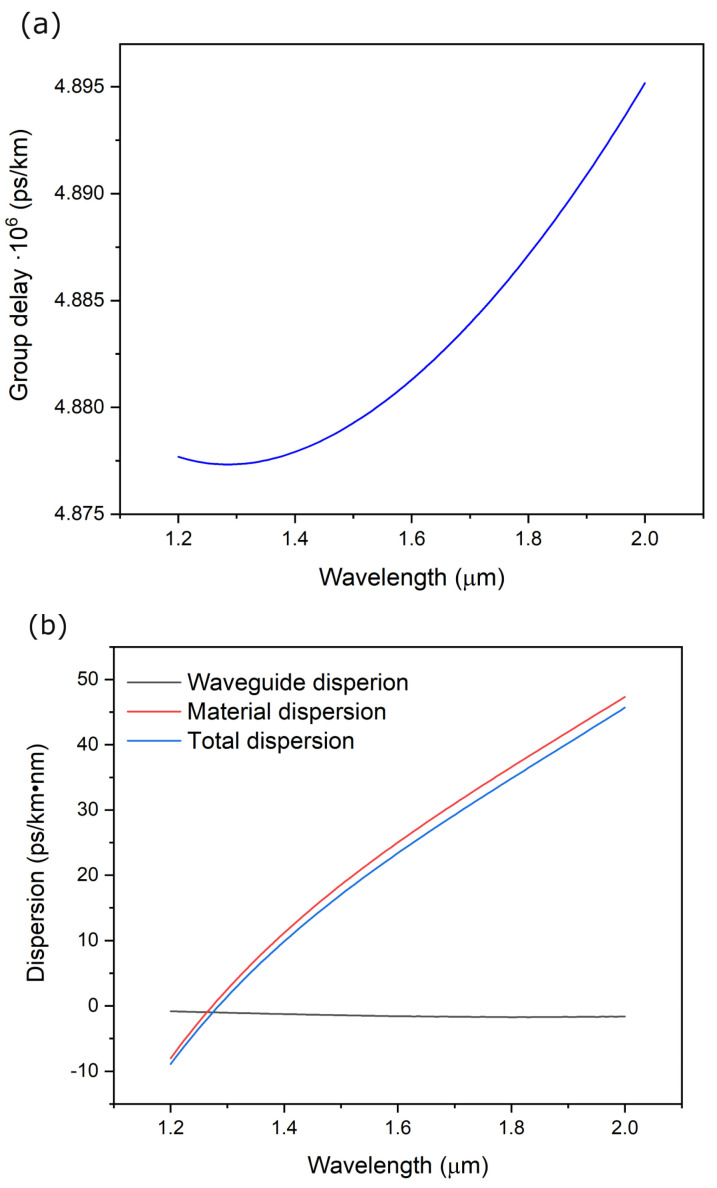
(**a**) The group delay of the presented construction of the optical fiber the preform and (**b**) the dispersion characteristic (numerical simulation).

Figure 5a,b shows the relationship between the group delay, the total dispersion, and its components (waveguide and material). The group velocity for wavelengths in the range of 1.55–2.0 µm varies in the range of 4.880 to 4.895 µs/km, and the value of total dispersion is in the range of 20.6–45.7 ps/km·nm. It is also worth emphasizing that the developed optical fiber design ensures single-mode propagation in the entire spectral range considered.

**Figure 6:**
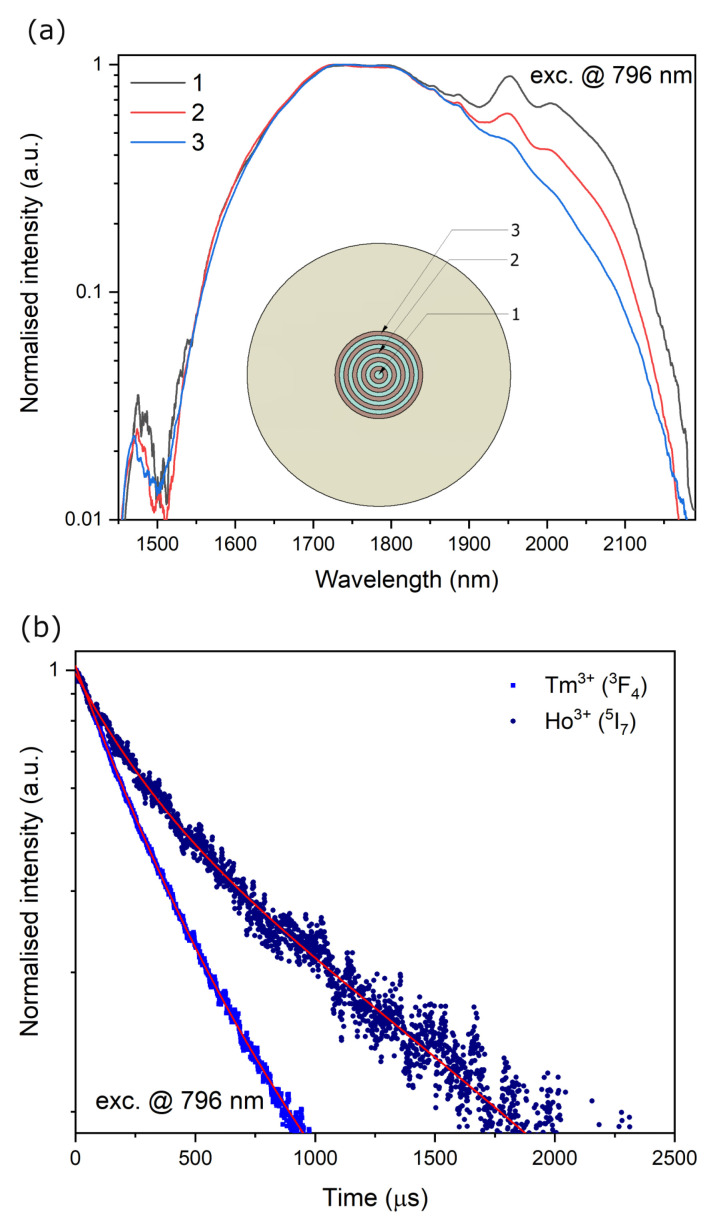
(**a**) The luminescence spectra of the optical fiber preform, inset: energy level of Tm^3+^/Ho^3+^ co-doped silica (ET-energy transfer) and (**b**) the decay time measurement of the fiber preform core excitation @ 796 nm monitoring @ 1800 nm (^3^H_4_, Tm^3+^) and 2050 nm (^5^I_7_, Ho^3+^).

The first stage of characterization of the properties of the preform was the measurement of the luminescence spectrum when the core area (illuminated over the entire surface) was excited with laser radiation with a wavelength of 796 nm. The recorded spectrum is shown in Figure 6a. Analyzing the recorded spectrum, we can notice two broad emission bands for thulium and holmium, typically observed in the glassy matrix resulting from the Stark splitting effect. The nearly uniform emission profile consists of the emission band of Tm^3+^ ions (resulting from the ^3^H_4_ →^3^H_6_ transition) and Ho^3+^ (^5^I_7_→^5^I_8_ one). The broad emission profile (FWHM = 415 nm @ 3 dB) confirms that it is possible to obtain broadband emission in optical fibers. Additionally, an emission spectrum near 1460 nm is visible, which is related to the cross-relaxation process of the neighboring thulium ions. The double exponential decay curve was used for the luminescence kinetics function analysis. The average lifetime was calculated using a double exponential decay fitting approximation (marked in Figure 6b using red color). The Tm^3+^ (^3^F_4_) and Ho^3+^ (^5^I_7_) decay time equals 526 µs (Adj. R-Square = 0.992) and 1071 µs (Adj. R-Square = 0.999), respectively. The double exponential fittings suggest the possible energy transfer processes or cross-relaxation exponentials that influence the shape of the excited state depopulation curves.

### 3.2. Optical Fiber Characterization

**Figure 7:**
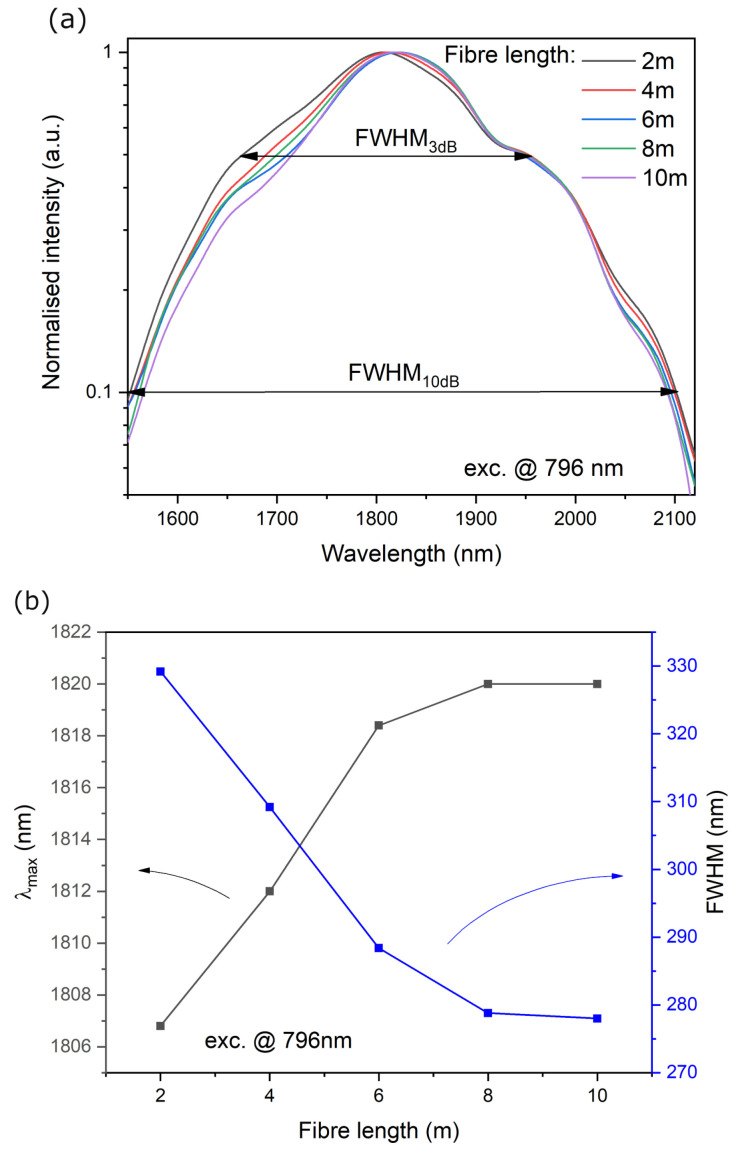
(**a**) The luminescence spectra of the fiber @ exc. 796 nm and (**b**) the FWHM and λmax vs. fiber length.

The double-clad single-mode optical fiber (18 μm core and 240 μm cladding) was drawn and characterized of the luminescence spectrum profile modification using the cut-back method @ exc. 796 nm. The analysis of the measured emission spectra along the optical fiber length in the range of 2–10 m showed that the half-width (FWHM_3dB_) of the profile varies in the range of 330–278 nm and λmax: 1806–1820 nm (Figure 7a,b). The 10-decibel luminescence band has also been determined and has the highest value for a fiber optic length of 2 m, which is FWHM_10dB_ = 549 nm. So far, many designs of broadband radiation sources have been developed based on the construction of silica fibers doped with thulium, holmium, and co-doped with thulium-holmium, obtaining broadband emission often also used to generate laser radiation with a tunable wavelength of emissions falling within the profile emissions of rare earth elements [29,42,57,58,59,60]. Additional broadening of the emission bandwidth is achieved by using ASE signals created in the forward and backward direction, e.g., obtaining 644 nm @ 10 dB bandwidth [30]. The narrowing of the luminescence spectrum in the optical fiber structure results from the phenomena of radiation reabsorption in its structure. It is also significantly narrower than the spectrum measured for the fiber optic preform. The calculated numerical aperture of the fiber core was NAc/cl = 0.054 (core/cladding). The applied refractive index of the polymer outer cladding was approximately n = 1.38, which allows the effective coupling of excitation radiation into the fiber structure (NA = 0.48). The fiber’s attenuation coefficient measured for 2nd transmission window was below 20dB/km. This structured optical fiber with a multi-ring core holds promise for use in novel optical radiation source designs.

## 4. Conclusions

The multi-ring core (Tm^3+^ and Tm^3+^/Ho^3+^ layers) LMA fiber fabricated using the MCVD-CDT system was presented and characterized. The low alumina content (Al_2_O_3_: 0.03wt%) was used for minimizing the refractive index modification and wide mode field propagation LP_01_ (MFD = 69.7 µm @ 2000 nm). The maximum concentration of lanthanide oxides in the fiber is as follows: Tm_2_O_3_: 0.18wt% and Ho_2_O_3_: 0.15wt%. The double-clad design of optical fiber emission spectra falling within the eye-safe spectral range of (1.55–2.10 µm) was presented. The variations in Full Width at Half Maximum (FWHM) and peak wavelength (λmax) vs. fiber length were considered.

## Data Availability

The data presented in this study are available on request from the corresponding author.
